# The effect of parity, breastfeeding history, and duration on clinical and pathological characteristics of breast cancer patients

**DOI:** 10.55730/1300-0144.5784

**Published:** 2023-11-18

**Authors:** Naziye AK, Zeynep TUZ, Esra AYDIN, Ferhat FERHATOĞLU, Murat SARI, Nail PAKSOY, İzzet DOĞAN, Anıl YILDIZ, Rian DİŞÇİ, Pınar Mualla SAİP

**Affiliations:** 1 Department of Medical Oncology, Faculty of Medicine, Demiroğlu Bilim University, İstanbul, Turkiye; 2 Department of Radiology, İstanbul Faculty of Medicine, İstanbul University, İstanbul, Turkiye; 3 Department of Medical Oncology, Recep Tayyip Erdogan University, Rize, Turkiye; 4 Department of Medical Oncology, Basakşehir Çam ve Sakura City Hospital, İstanbul, Turkiye; 5 Department of Medical Oncology, Marmara Faculty of Medicine, Marmara University, İstanbul, Turkiye; 6 Department of Medical Oncology, Tekirdağ İsmail Fehmi Cumalıoğlu City Hospital, Tekirdağ, Turkiye; 7 Department of Medical Oncology, İstanbul Faculty of Medicine, İstanbul University, İstanbul, Turkiye; 8 Faculty of Biostatistical Science, Beykent University, İstanbul, Turkiye

**Keywords:** Breastfeeding, delivery, breast cancer, stage, lactation, breastfeeding duration

## Abstract

**Background/aim:**

The study is aimed to determine the relationship between the delivery and breastfeeding history of the patients and the clinicopathological properties of breast cancer.

**Materials and methods:**

A questionnaire was utilized for the study, which included the age of diagnosis, the number of children at the time of diagnosis, the age of the children, and the breastfeeding period of each child.

**Results:**

The study included 828 patients. The median age at diagnosis was 47 years for parous women and 42 years for nonparous women (p < 0.001). The tumor size of the patients diagnosed within the breastfeeding period was significantly larger compared to the other patients. Estrogen and progesterone receptor positivity were lower in patients diagnosed during breastfeeding. Additionally, the mean number of positive lymph nodes, dissected lymph nodes, and positive lymph node/dissected lymph node ratio in parous and breastfed patients with a nonmetastatic disease were statistically significantly higher in multivariable analysis than those patients who were nulliparous and have not breastfed.

**Conclusion:**

Breast cancer is seen at a later age in patients who are parous than those who have never given birth. Patients who are parous and have breastfed tend to present with a higher stage of the disease.

## 1. Introduction

Breast cancer is observed to be the most frequent type of cancer among women, and it is also the primary cause of mortality for cancer-related deaths in women. Breast cancer risk has been shown to identify with various factors such as age, sex, race, genetic factors, hormonal factors, family history, ionizing radiation, as well as lifestyle traits [1–3]. It is evident from the literature that breastfeeding for at least six months has a protective effect on breast, ovarian, and endometrial cancer [4–6]. Moreover, women with primary breast cancer have a better survival rate if total breastfeeding is longer than six months [7]. The protective factor of breastfeeding suggests a more pronounced effect on hormone receptor-negative breast cancers [8]. The results of a meta-analysis depicted an inverse relationship between breastfeeding and breast cancers that were positive or negative for both hormone receptors (ER+ and PR+; or ER− and PR−), but it did not take HER2 into consideration [9].

The studies investigating breastfeeding’s relation with breast cancer subgroups only considered characteristics such as histological type and estrogen receptor status. Interestingly, only Butt et al. found a trend towards more grade III tumors and high Ki67 expression with increasing duration of breastfeeding, but their results did not show any correlation about molecular subtypes [10]. Our study design was constructed to evaluate a wide number of parameters of breast cancer patients’ characteristics, such as stage factors, treatment modalities, and pathologic properties. On the other hand, it must be stated that the effect of various differing breastfeeding patterns and the duration of breastfeeding on patients who have been already diagnosed with the disease is not well determined. Also, studies about the relationship between breastfeeding history/duration and breast cancer subtype are sparse and inconsistent for Turkish women. We aimed with this study to determine the relationship between histopathological features of breast cancer and pregnancy or breast cancer with breastfeeding history in the Turkish patient population.

## 2. Materials and methods

This single-center-based study was conducted as a cross-sectional study. It included female patients who were diagnosed with breast cancer and who were admitted to outpatient clinics for either follow-up and/or active treatment visits for the disease between the years of 2018 and 2019. The institutional review board and the Ethics Committee approved the study. A questionnaire that included the age at diagnosis, the number of children at the time of diagnosis, the age of the children, and the breastfeeding period of each child were applied to samplings (Supplementary Material 1). Information was provided to the patients about the planned study, and their informed consent was obtained before the questionnaire was applied. We collected information related to the reference year, defined as the histological date of the diagnosis. After starting the questionnaire, patients reported remarkable findings, such as the condition of limited breastfeeding from the breast with cancer, even before the diagnosis. Subsequently, approximately half of the patient group was asked a question about the dominant breast that the patient used for breastfeeding purposes. After the data were collected, we calculated the lifetime duration of the breastfeeding and the time passed since the last breastfeeding activity.

Additionally, clinical data such as menopause status, height-weight information at the time of diagnosis, histopathological features of the tumor, stage of the disease, and the first treatment modality were noted. Terminologies about parous, primiparous, multiparous, and nulliparous are determined as having given birth to one or more children, having given birth to one child, having given birth to multiple children, and not having given birth to any children, respectively. Hospital records were used to collect the necessary information related to molecular breast cancer subgroups that were previously determined by utilizing immune staining for identifying ER, PR, and HER2 molecules. ER and PR positivity are defined according to immunohistochemical staining of tumoral tissue of more than 1%. HER2 expression was accepted as negative in patients with Score 0 and Score 1, and positive in patients with score 3, according to membrane positivity ratio in immunohistochemical staining. In situ hybridization of HER2 gene expiration was requested from all patients with score 2 to determine the positive or negative status. Radiologic and clinical staging was done for local disease with ultrasonography, mammography, breast MR imaging, and for distant evaluation with computed tomography of the thorax and abdomen. Pathological staging was performed in accordance with the American Joint Committee on Cancer (AJCC)-8th criteria after surgery. The study endpoint was to examine the relation between the clinical and tumor-related characteristics and history of breastfeeding and the parity of the patients with breast cancer.

SPSS 20.0 (IBM, Armonk, New York) software was utilized for the statistical analyses of the study data. The number, and the percentages for categorical variables, mean, standard deviation, median, minimum, and maximum values of numerical variables were calculated for descriptive statistics. Continuous variables were compared using the Kruskal-Wallis H and the Mann-Whitney U tests when the data did not appear to have a normal distribution. In addition, the Chi-square test was used to compare the categorical subgroups. Multivariable analysis for clinical factors that may compromise the patients’ positive/resected lymph node ratio was analyzed with a logistic regression test. A p-value of <0.05 was considered statistically significant.

## 3. Results

A total of 828 patients were included in the study. The median age of patients at the time of diagnosis was 46 (Range: 23 – 83) years, and the median age at the time of first delivery was 24 (Range: 15–45) years for all populations. The median age at diagnosis was 47 years for parous women and 42 years for those who were nonparous. Nonparous women with breast cancer were diagnosed at an earlier age which was statistically significant (p < 0.001). Similarly, those who delivered once were diagnosed at an earlier age than those who delivered two or more (p < 0.001). The median tumor size was 2.5 (0.2–16) cm. The tumor size of patients diagnosed within the time of breastfeeding was larger than others (p = 0.010). The median number of children for parous patients at the time of diagnosis was 2 (range: 1–8). In the whole group, the birth rate was 89.5%, and parous women’s breastfeeding rate was 94.7%. Demographic data, parity, and breastfeeding characteristics of the patients are summarized in Table 1, and pathological characteristics of the disease are summarized in Table 2. However, the patients reported that they had limited breastfeeding from the breast with cancer during the questionnaire; the dominant breast for breastfeeding and the tumoral site were not negatively correlated. Also, there was no correlation between the histological subtype of the tumor, breastfeeding time or time after breastfeeding, and delivery history. Although it did not reach statistical significance, the hormone-positive disease was observed more frequently in parous patients and patients with breastfeeding, with rates of 78.2% vs. 84.1% (p = 0.214) and 83.6% vs. 88.5% (p = 0.121), respectively. For the patients diagnosed during their period of breastfeeding, it was observed that the ER and PR positivity rates were relatively lower than others (median for ER positivity: 37.5 vs. 80, p = 0.045; median for PR positivity: 0 vs 30, p = 0.001). Also, we found that patients with three or more children had the same percent of ER status as others, but relatively low PR status, which was found to be significant (p = 0.047), suggesting a tendency toward luminal B subtype (Table 3).

According to the patients’ parity and breastfeeding history, there was a statistically significant difference between the disease stage at the time of disease presentation. The N1-N2-N3 ratios of parous and breastfeeding patients were higher than those not breastfeeding, while the N0 ratio was observed to be lower (p < 0.001). The mean number of positive, dissected lymph nodes, and the positive lymph node/dissected lymph node ratio among the patients who are parous and have breastfed, were found to be statistically significantly higher than those who have not breastfed (p < 0.001, p = 0.039, p = 0.003) (Table 4). Similarly, positive lymph count, dissected number of lymph nodes, as well as positive/dissected lymph node ratio had a statistically significant correlation with the number of births (p < 0.001, p = 0.015, p < 0.001) (Table 5). A significant (r = 0.142/p < 0.0001) positive correlation was observed between breastfeeding duration and the number of positive lymph nodes. Significant efficacy of BMI, inflamatory status., T stage, parity, breastfeeding, total breastfeeding month and histological subtype of triple negative were observed as predictive factor for patients with positive lymph node in the univariable model (p < 0.05). Also, significant-independent (p < 0.05) efficacy of T stage and breastfeeding was observed in predicting patients with positive lymph nodes and positive/dissected lymph node ratio in the multivariable model (Supplementary Table 1).

## 4. Discussion

The present study has evaluated the association between disease characteristics of patients with breast cancer and their reproductive history, which consists of the number of deliveries and the time of delivery, as well as different breastfeeding behaviors. Patients who had given birth and who had breastfed their child were presented with a higher rate of positive lymph nodes and, consequently, with a higher nodal stage. Despite the other studies that have shown an association between hormone and HER2 receptor expression of tumors and the reproductive history of patients, we could not show such an association in our study.

Pregnancy has a dual effect on breast cancer development, depending on age at first delivery. Younger mothers mostly derive protection benefits from pregnancy. Nevertheless, mothers older than 35 have an increased risk of breast cancer [11]. Although pregnancy-associated breast cancer (PABC) is defined as breast cancer during pregnancy or within postpartum 12 months, the peak incidence of breast cancer after delivery occurs approximately six years postpartum [12]. PABC was found to be presented with a higher nodal stage in previous studies [13]. Another study also showed higher mortality in patients diagnosed after pregnancy; interestingly, which was not just after pregnancy but peaking two years after diagnosis and continuing until ten years later [14]. Based on these studies, it is thought that pregnancy’s changes in the breast tissue have long-term effects. Also, after pregnancy, lactation is another modifying factor in breast tissue.

At the peak lactation period, the mammary gland functions with a higher metabolism speed. Prevalent transformations in the metabolism of various tissues, which are required to ensure plentiful sources of nutrients, occur during the milk-production process [15]. Subsequently, a process called involution that is classified into two distinct phases, and is triggered after a short time following weaning, due to milk stasis and the reduction of lactogenic hormones [16]. It starts with the initial and reversible phase, which involve the dissolution of physio-chemical adaptations and then apoptosis. Subsequently, these cells acquire an inflammatory profile. The second phase of involution involves an irreversible remodeling and restructuring process through a proteolytic degradation of the basement membrane, accompanied with the remodeling of the mammary gland, with subsequent epithelial cell replacement due to the differentiation of adipocyte and proliferation. It is interesting to note that the mammary gland changes during its postlactational period resemble the wound-healing process and a tumor microenvironment [17]. In fact, it has been thought that macrophages could be influential in enticing postpartum breast cancer [18]. Similar to this inflammation theory, another clinical study shows that patients with mastitis are prone to develop breast cancer. However, the authors of this study did not evaluate the stage of the disease, and the study was only based on risk analysis [19].

Borges et al. have speculated that this involution plays a role in the generation of more aggressive breast cancers within the decade following childbirth [20]. In addition, other researchers have provided evidence that cells from very early-stage tumors cells can be released into the stroma during involution, where they have access to the vasculature [21, 22]. Lyons et al. demonstrated that postpartum patients tend to be present with a greater number of lymph node metastases than age-matched nulliparous controls. However, they did not evaluate the breastfeeding history of their study group, and our study may be able to add a comment to their hypothesis. Furthermore, they evaluated only young patients (<40 years) and patients who were diagnosed within two years of their delivery [22]. Our study showed that delivery and breastfeeding might have a long-term provocative effect on lymph node metastasis in breast cancer patients. However, our parous patient population showed a breastfeeding rate of 94.7%. Only 39 patients have no breastfeeding history in the parous group. Since our study has very low statistical power to dissociate the effect of parity or breastfeeding on breast cancer patients, we could not able to attribute these results to a single process. Extracellular and angiogenetic differences that developed after parity and lactation might have resulted in earlier and more promoted tumoral spreading. Considering the higher nodal stage of patients with parity and breastfeeding rather than tumoral size in our study, there could be a different mechanism about tumoral invasiveness rather than the generation of the tumor or its local growth.

It is well documented that metalloproteinase (MMP) enzymes, which are a family of proteases, play a major role in mammary gland remodeling [18, 23]. In their mechanism, MMPs create their influence primarily by modifications in the extracellular matrix. Furthermore, it has been stated that under some physiological and pathological conditions, MMP and cathepsins can affect intracellular proteins, as these can contribute to an increase in cell invasion [24]. In addition, it has been stated that enhanced alveologenesis and angiogenesis during lactation might contribute to the enhanced potential of spreading of the tumor cells to lymph nodes [25]. This hypothesis needs to be evaluated more appropriately in the analysis of breast cancer tissue.

Even though the protective effect of parity is pronounced for breast cancers with ER+ and/or PR+; the effect on ER−/PR− or triple-negative disease is conflicting with different studies that state increased risk [26, 27], or no association [28]. On the other hand, breastfeeding lowers the breast cancer risk by suppressing ovulation and promoting breast epithelial restoration. The protective effect of breastfeeding is thought to be for younger patients and for hormone-negative tumors [29]. John et al. stated that parous women without breastfeeding have a two-fold increased risk for triple-negative disease compared to nulliparous women. The results of this study, which was also supported by the results of a meta-analysis, showed that breastfeeding was related to a 10% decrease in the risk of breast cancers, which were negative for both ER and PR in parous women [30].

Additionally, a longer duration of breastfeeding results in a more pronounced protective effect [31]. The mechanism that is responsible for breastfeeding’s protective effect against hormone-negative disease needs to be investigated further on a molecular basis. Recently, Mejri et al. reported the association of a longer duration of breastfeeding with inflammatory breast cancer, similar to previous studies [32, 33]. Interestingly, another study conducted with eighty patients reported that the absence of lactation showed a tendency to larger tumors, more axillary lymph node involvement, and distant metastases; however, the results were statistically insignificant. In the same study, higher proliferation was shown by immunohistochemical expression of Ki-67 [34]. All of the previously mentioned studies show that there is no consensus about the effect of breastfeeding on molecular subtypes of the disease. Also, we were not able to show any correlation of parity or breastfeeding history with the histological subtype of inflammatory disease; however, we showed that hormone receptor rates were relatively lower in patients diagnosed within the duration of lactation and with lower than three deliveries. This result needs to be confirmed with studies that consist of a greater number of patients. We also have to state that our study population was from unselected polyclinic breast cancer patients that consists of a much higher rate of parity (89.5%) and breastfeeding (84.8% for all groups, 94.7% for the parous group) than all of the previously mentioned studies. Therefore, the detection of such a relation is relatively difficult for our patient population.

Several limitations should be mentioned regarding this study. First of all, it was a retrospective study and conducted only on patients with breast cancer, so there was no control group to use as a reference. Also, since there are cultural differences in our country as compared to the Western countries that have produced the studies that we have mentioned in our literature review, there was a small number of patients who were nulliparous and parous but who did not engage in breastfeeding in our study. So, this study is not able to show stage differences due to delivery and breastfeeding in the breast cancer population. On the other hand, the stages of the patients are recorded from pathological reports, so the effect of neoadjuvant treatment on pathological staging could not be directly evaluated. Our study might be a starting point for delivery, and breastfeeding changes might constitute an inflammatory status that leads to accelerated lymphatic drainage. Thus, molecular studies that evaluate the differences between breastfeeding and parous women’s breast and tumoral microenvironment might show the causes or biological basis of this clinical presentation.

## 5. Conclusion

Breast cancer is seen at a later age in patients who have given two or more births than those who have never given birth or those who have given only a single birth. The presence of delivery and breastfeeding did not affect the histological features of the tumor. However, patients who were diagnosed within the duration of breastfeeding, and those who have more than three children, were observed to have a lower hormone receptor positivity rate. Also, parous and breastfed patients tend to have a higher number of positive lymph nodes. This study and further studies on the subject can pave the way for better understanding and the treatment of breast cancer in a longer postpartum period.

## Supplementary Data

**Supplementary Table 1:** Analyzed clinical factors on positive/dissected lymph node ratio.

	Univariable model					Multivariable model				
	OR	% 95 CI			p	OR	% 95 CI			p
BMI	1.04	1.01	-	1.07	***0.012***					
Inflamatory status	0.12	0.01	-	0.89	***0.038***					
T Stage	2.87	2.21	-	3.73	***0.000***	0.19	0.10	-	0.36	***0.000***
Parity	0.61	0.47	-	0.79	***0.000***					
Breatsfeeding	0.35	0.23	-	0.53	***0.000***	0.20	0.11	-	0.36	***0.000***
Total breastfeeding month	1.01	1.00	-	1.02	***0.001***					
Hıstologıcal subtype	0.77	0.64	-	0.92	***0.005***					
Logistic regression (Forward LR)										



## Figures and Tables

**Table 1:** Patient characteristics.

**Age** n (%)	≤50 years	511 (63.7)
	>50 years	291 (36.3)
**Parity** n (%)	Primiparous	199 (24.0)
	Multiparous	542 (65.5)
	Nulliparous	87 (10.5)
**Breastfeeding** n (%)	Yes	702 (84.8)
	No	126 (15.2)
**Breastfeeding in parous patients** n (%)	Yes	702 (94.7)
	No	39 (5.3)
**Subgroups according to breastfeeding** n (%)	None	126 (15.2)
	≤12 months	216 (26.1)
	12–24 months	155 (18.7)
	≥24 months	331 (40.0)
**Subgroups according to time from last breastfeeding** n (%)	≤1 year	35 (5.0)
	1 to 2 years	26 (3.7)
	2 to 5 years	59 (8.4)
	5 to 10 years	91 (13.0)
	10 to 20 years	228 (32.5)
	≥20 years	263 (37.4)

Min: Minimum, Max: Maximum, SD: Standard deviation

**Table 2:** Disease characteristics.

		n	%
**Diagnosis during pregnancy**	Yes	13	1.6
	No	815	98.4
**Diagnosis during breastfeeding**	Yes	22	2.7
	No	806	97.3
**Location**	Right breast	202	50.8
	Left breast	186	46.7
	Bilateral	10	2.5
**Tumor diameter (Cm)** median (Range)		2.50 (0.2–13)	
**Clinical stage**	Stage 1	92	11.6
	Stage 2	325	40.9
	Stage 3	336	42.3
	Stage 4	42	5.3
**First treatment modality**	Adjuvant	410	49.5
	Neoadjuvant	376	45.4
	Metastatic	42	5.1
**T stage** *	T1	278	36.4
	T2	413	54.1
	T3	57	7.5
	T4	15	2.0
**N stage** *	N0	301	40.0
	N1	284	37.8
	N2	115	15.3
	N3	52	6.9
**Histological subtypes**	HR positive-HER2 positive	116	15.3
	HR positive-HER2 negative	483	63.6
	HR negative-HER2 positive	71	9.3
	Triple negative	90	11.8
**Hormone status**	HR positive	615	78.8
	HR negative	165	21.2
**HER2 status**	Positive	194	24.6
	Negative	596	75.4

Cm: Centimeter, SD: Standard deviation, T: Tumor, N: Node, ER: Estrogen receptor, PR: Progesterone receptor, HR: Hormone receptor

* For operated patients

**Table 3:** Relation of reproductive properties and hormone receptor positivity.

		ER positivity (%)	PR positivity (%)
		Mean ± SD (median, range)	Mean ± SD (median, range)
**Parity**	Yes	60.1 ± 38.0 (80, 0–100)	37.3 ± 36.8 (30, 0–100)
	No	61.0 ± 37.4 (80, 0–100)	35.5 ± 34.0 (30, 0–100)
	**p-value**	0.564	0.924
**Number of delivery**	≤2	61.7 ± 36.9 (80, 0–100)	38.7 ± 37.0 (30, 0–100)
	≥3	56.8 ± 39.9 (80, 0–100)	33.6 ± 35.3 (20, 0–100)
	**p-value**	0.273	**0.047**
**Breastfeeding**	Yes	59.5 ± 38.2 (80, 0–100)	36.9 ± 36.8 (25, 0–80)
	No	63.6 ± 36.2 (80, 0–100)	38.3 ± 35.2 (30, 0–100)
	**p-value**	0.226	0.434
**Diagnosis during breastfeeding**	Yes	42.7 ± 43.8 (37, 0–100)	14.1 ± 26.8 (0, 0–80)
	No	62.1 ± 37.0 (80, 0–100)	38.3 ± 36.5 (30, 0–100)
	**p-value**	**0.045**	**0.001**
**Duration of breastfeeding**	<24 months	60.4 ± 37.3 (80, 0–100)	38.1 ± 36.7 (30, 0–100)
	≥24 months	60.1 ± 38.7 (80, 0–100)	36.1 ± 36.2 (25, 0–100)
	**p-value**	0.845	0.256

**Table 4:** Tumor characteristics of patients according to breastfeeding properties.

		Lactation								
		None		≤12 m		12–24 m		≥24 m		
		n	%	n	%	N	%	n	%	p
**Clinical Stage**	Stage 1	16	12.7	28	13.6	17	11.8	31	9.9	0.382
	Stage 2	53	42.1	85	41.3	55	38.2	129	41.2	
	Stage 3	46	36.5	81	39.3	68	47.2	139	44.4	
	Stage 4	11	8.7	12	5.8	4	2.8	14	4.5	
**Pathological stage** *	Stage 1	31	27.4	55	28.4	34	24.1	61	20.5	0.322
	Stage 2	62	54.9	98	50.5	70	49.6	165	55.4	
	Stage 3	20	17.7	41	21.1	37	26.2	72	24.2	
**T Stage** *	T1	43	37.7	80	40.2	53	37.1	102	33.8	0.716
	T2	61	53.5	100	50.3	79	55.2	169	56.0	
	T3	10	8.8	14	7.0	9	6.3	23	7.6	
	T4	0	0.0	5	2.5	2	1.4	8	2.6	
**N stage** *	N0	69	60.5	74	38.1	53	37.6	102	34.2	**<0.001**
	N1	30	26.3	84	43.3	51	36.2	118	39.6	
	N2	9	7.9	24	12.4	24	17.0	57	19.1	
	N3	6	5.3	12	6.2	13	9.2	21	7.0	
**Histological subtypes**	HR Positive-HER2 Negative	80	67.2	125	64.8	87	63.0	188	62.0	0.948
	HR Negative-HER2 Positive	8	6.7	17	8.8	14	10.1	31	10.2	
	Triple Negative	11	9.2	25	13.0	15	10.9	37	12.2	
	HR Positive-HER2 Positive	20	16.6	26	13.5	22	15.9	47	15.5	
**Hormone status**	Positive	101	84.2	156	78.0	112	78.9	242	77.8	0.509
	Negative	19	15.8	44	22.0	30	21.1	69	22.2	
**HER2 status**	Positive	29	23.4	44	22.1	37	25.9	82	25.9	0.762
	Negative	95	76.6	155	77.9	106	74.1	235	74.1	
**HER2 positive subgroup**	HR positive	20	71.4	26	60.5	22	61.1	47	60.3	0.750
	HR negative	8	28.6	17	39.5	14	38.9	31	39.7	
**First treatment modality**	Adjuvant	49	38.9	99	48.1	76	52.1	158	49.8	0.162
	Neoadjuvant	66	52.4	95	46.1	66	45.2	145	45.7	
	Metastatic	11	8.7	12	5.8	4	2.7	14	4.5	
T Diameter (Cm)* Mean ± SD (Median)		3.0 ± 1.9 (2.5)		2.9 ± 1.9 (2.3)		2.8 ± 1.5 (2.6)		3.0 ± 1.7 (2.5)		0.611
Positive N* **(n)** Mean ± SD (Median)		1.4 ± 2.8 (0)		2.1 ± 3.6 (1)		2.6 ± 4.0 (1)		2.8 ± 4.0 (1)		**<0.001**
Dissected N* **(n)** Mean ± SD (Median)		8.1 ± 7.7 (4)		9.1 ± 7.7 (6)		9.7 ± 7.3 (10)		10.0 ± 7.7 (8)		**0.039**
Positive/dissected N ratio* Mean ± SD (Median)		0.13 ± 0.24 (0)		0.20 ± 0.32 (0.08)		0.21 ± 0.26 (0.10)		0.23 ± 0.30 (0.13)		**0.003**
**Subgroup analysis**										
	**None vs. ≤12 m**	**None vs. 12**–**24 m**		**None vs. ≥24 m**		**≤12 vs. 12**–**24 m**		**≤12 vs. ≥24 m**		**12–24 m vs. ≥24 m**
	**p**	**p**		**p**		**p**		**p**		**p**
**Positive N** *	**0.002**	**<0.001**		**<0.001**		0.199		0.129		0.909
**Dissected N** *	0.147	**0.013**		**0.021**		0.156		0.344		0.508
**Positive/dissected N Ratio** *	**0.006**	**0.001**		**<0.001**		0.379		0.132		0.668

* Operated patients,

T: Tumor, N: Node, HR: Hormone receptor

**Table 5:** Tumor characteristics of patients according to parity groups.

		Parity						
		Primiparous		Multiparous		Nulliparous		
		n	%	n	%	n	%	p
**Clinical stage**	Stage 1	18	9.4%	61	11.8%	13	14.9%	0,108
	Stage 2	75	39.1%	212	41.1%	38	43.7%	
	Stage 3	88	45.8%	221	42.8%	31	35.6%	
	Stage 4	11	5.7%	22	4.3%	5	5.8%	
**Pathological stage** *	Stage 1	47	26.0%	111	22.5%	23	30.3%	**0.040**
	Stage 2	93	51.4%	260	52.6%	46	60.5%	
	Stage 3	41	22.7%	123	24.9%	7	9.2%	
**T stage** *	T1	79	42.9%	170	33.9%	29	37.7%	0.310
	T2	91	49.5%	280	55.8%	42	54.5%	
	T3	10	5.4%	41	8.2%	6	7.8%	
	T4	4	2.2%	11	2.2%	0	0.0%	
**N stage** *	N0	66	36.5%	184	37.2%	51	66.2%	**<0.001**
	N1	70	38.7%	193	39.1%	21	27.3%	
	N2	32	17.7%	80	16.2%	3	3.9%	
	N3	13	7.2%	37	7.5%	2	2.6%	
**Histological subtypes**	HR positive-HER2 positive	32	17.4	69	13.9	32	17.4	0.648
	HR positive-HER2 negative	116	63.0	314	63.4	116	63.0	
	HR negative-HER2 positive	14	7.6	52	10.5	14	7.6	
	Triple negative	22	12.0	60	12.1	22	12.0	
**Hormone status**	HR positive	153	81.0	393	77.2	153	81.0	0.259
	HR negative	36	19.0	116	22.8	36	19.0	
**HER2 status**	Positive	46	24.7	127	24.5	46	24.7	0.997
	Negative	140	75.3	392	75.5	*140*	75.3	
**HER2 positive subgroup**	HR positive	32	69.6	69	57.0	32	69.6	0.148
	HR negative	14	30.4	52	43.0	14	30.4	
**First treatment modality**	Adjuvant	73	37.8	274	52.6	37	42.5	0.243
	Neoadjuvant	109	56.5	225	43.2	41	47.1	
	Metastatic	11	5.7	22	4.2	9	10.4	
**T diameter (Cm)** Mean ± SD (Median)		2.7 ± 1.5 (2.3)		3.0 ± 1.8 (2.5)		2.8 ± 1.6 (2.5)		0.135
**Positive N (n)** Mean ± SD (Median)		2.5 ± 3.6 (1)		2.5 ± 3.9 (1)		0.7 ± 1.6 (0)		**<0.001**
**Dissected N (n)** Mean ± SD (Median)		9.8 ± 7.8 (8)		9.6 ± 7.6 (8)		7.3 ± 7.3 (4)		**0.015**
**Positive/dissected N ratio** Mean ± SD (Median)		0.22 ± 0.27 (0.13)		0.21 ± 0.31 (0.10)		0.10 ± 0.22 (0)		**<0.001**

* Operated patients,

T: Tumor, N: Node, HR: Hormone receptor
