# Supplementary figures and images for: The effect of parity, breastfeeding history, and duration on clinical and pathological characteristics of breast cancer patients
Source: Turk J Med Sci. 2023 Nov 18;54(1):229–38. doi: 10.55730/1300-0144.5784 (PMC11031182; doi:10.55730/1300-0144.5784)

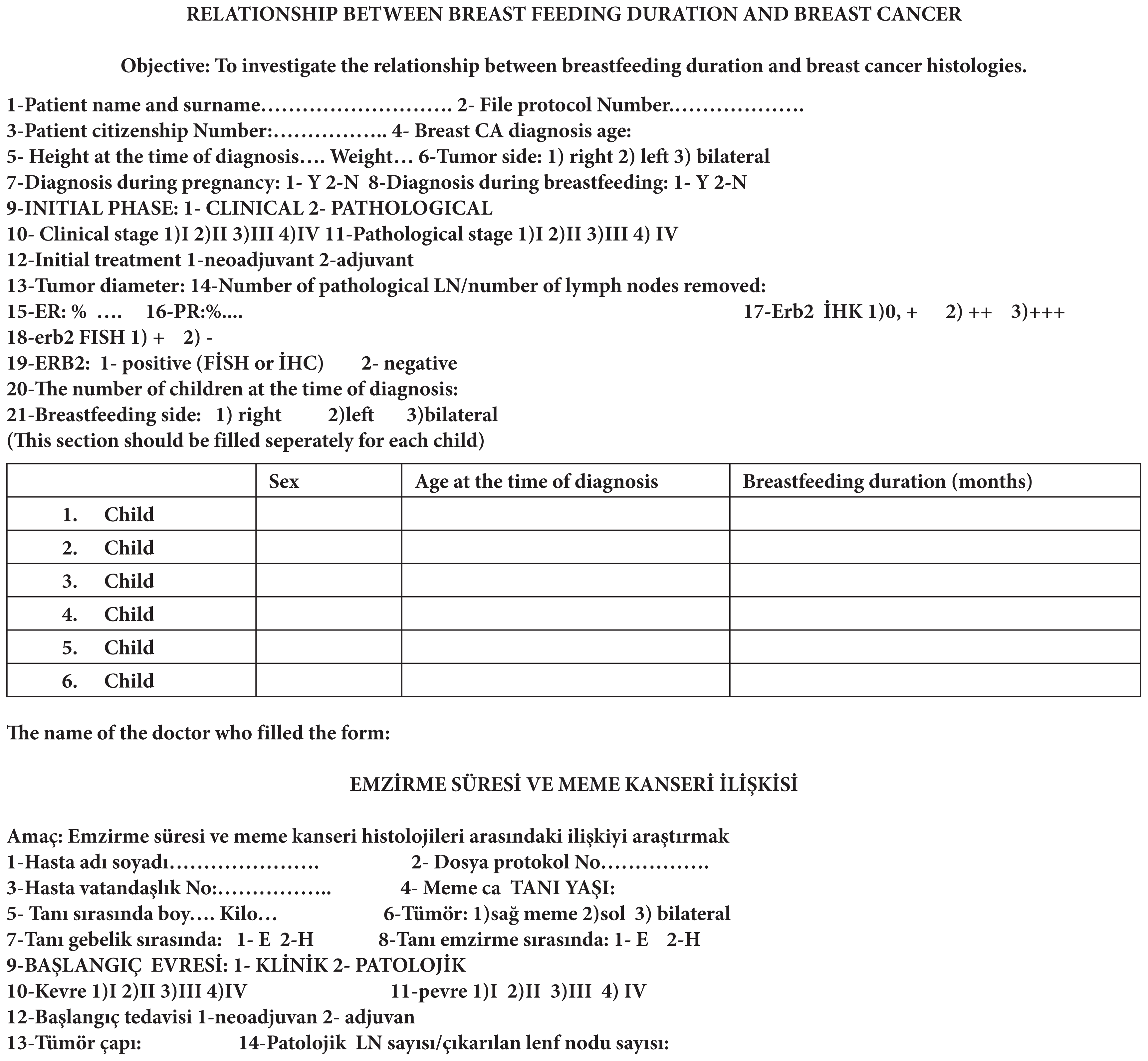

Supplement: Supplementary file 1 [file tjmed-54-01-0229s1a.tif]

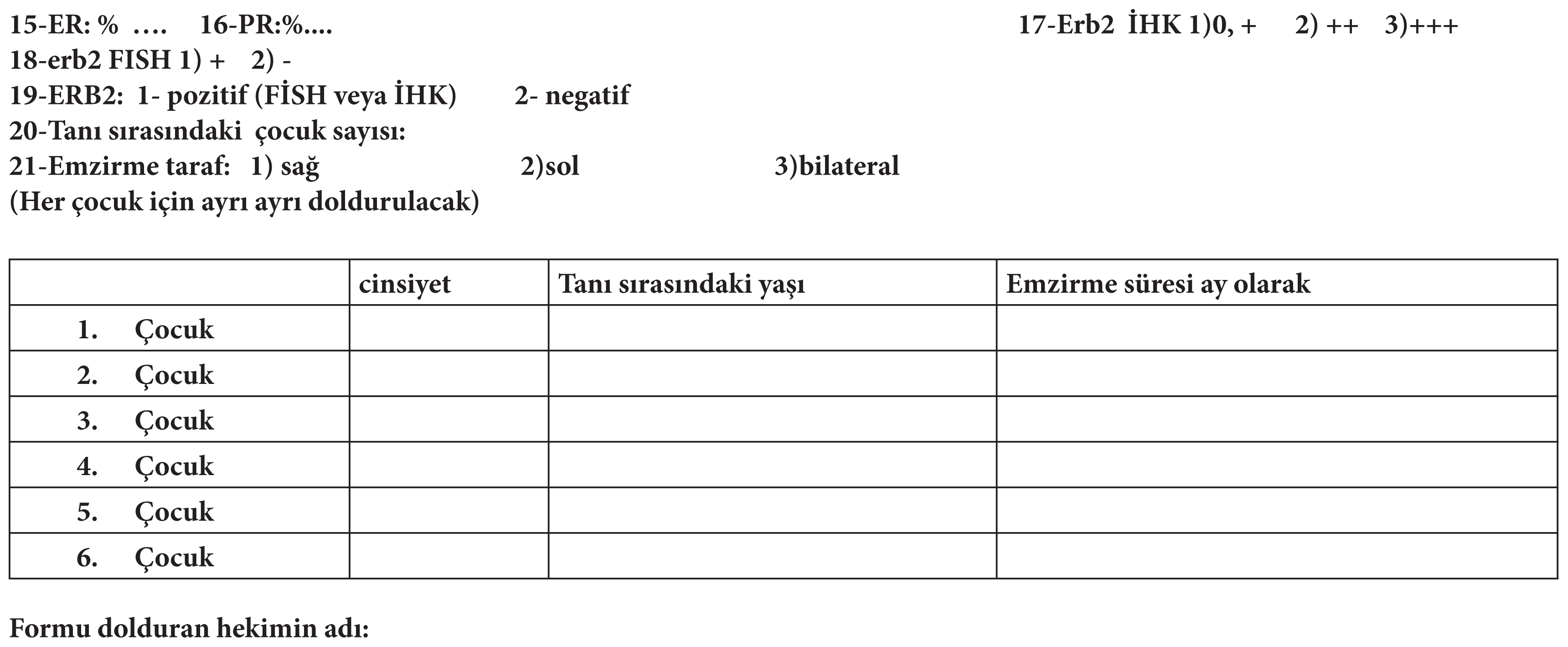

Supplement: Supplementary file 2 [file tjmed-54-01-0229s1b.tif]
